# Binge eating disorder, frequency of depression, and systemic inflammatory state in individuals with obesity – A cross sectional study

**DOI:** 10.20945/2359-3997000000489

**Published:** 2022-06-27

**Authors:** Nelson do Rosário Caldas, Valeria Bender Braulio, Marco Antônio Alves Brasil, Valeria Cristina Soares Furtado, Denise Pires de Carvalho, Ervin Michelstaedter Cotrik, Joana Rodrigues Dantas, Lenita Zajdenverg

**Affiliations:** 1 Universidade Federal do Rio de Janeiro Serviço de Psiquiatria e Psicologia Médica Rio de Janeiro RJ Brazil Universidade Federal do Rio de Janeiro, Serviço de Psiquiatria e Psicologia Médica, Rio de Janeiro, RJ, Brasil; 2 Universidade Federal do Rio de Janeiro Serviço de Nutrologia Rio de Janeiro RJ Brazil Universidade Federal do Rio de Janeiro, Serviço de Nutrologia, Rio de Janeiro, RJ, Brasil; 3 Universidade Federal do Estado do Rio de Janeiro Hospital Universitário Gaffrée e Guinle Rio de Janeiro RJ Brazil Universidade Federal do Estado do Rio de Janeiro, Hospital Universitário Gaffrée e Guinle, Rio de Janeiro, RJ, Brasil; 4 Universidade Federal do Rio de Janeiro Instituto de Biofísica Carlos Chagas Filho Rio de Janeiro RJ Brazil Universidade Federal do Rio de Janeiro, Instituto de Biofísica Carlos Chagas Filho, Rio de Janeiro, RJ, Brasil

**Keywords:** Binge eating disorder, obesity, depression, leptin, inflammation

## Abstract

**Objective:**

Binge eating disorder (BED) is the most prevalent eating disorder in individuals with obesity. Its association with factors that control hunger and satiety has not yet been elucidated. We evaluated whether levels of inflammatory markers, frequency of psychiatric comorbidities, and appetite-related hormones levels differ between individuals with obesity with and without BED.

**Materials and methods:**

The Structured Clinical Interview for Diagnostic and Statistical Manual of Mental Disorders-5 – Clinician Version (SCID-5-CV), Binge Eating Scale, and Hospital Anxiety and Depression Scale were evaluated in 39 individuals with obesity. Plasma levels of C-reactive protein (CRP), tumor necrosis factor-alpha (TNF-α), interleukin-6 (IL-6), leptin, ghrelin, and glucagon-like peptide-1 (GLP-1) were measured.

**Results:**

Individuals of the BED group exhibited significantly higher percentages of altered eating patterns (hyperphagia, bingeing, post-dinner eating, feeling “stuffed”, and emotional eating), higher depressive symptom scores and levels of leptin, CRP, and TNF-α, compared to those from the non-BED group. Logistic regression showed that BED was independently associated with depressive symptoms and CRP levels.

**Conclusions:**

Individuals with obesity and BED showed greater psychiatric comorbidity, worse eating patterns and worse inflammatory profile than those without BED. BED should be assessed as an indicator of clinical severity in patients with obesity.

## INTRODUCTION

Binge eating disorder (BED) was recognized as a feeding and eating disorder diagnostic entity in the Diagnostic and Statistical Manual of Mental Disorders, 5^th^ Edition (DSM-5), from the American Psychiatric Association ( 1 ). BED is characterized by recurrent episodes of binge eating – eating an unusually large amount of food compared to what one would eat within a certain period under similar circumstances – associated with a loss of self-control when eating and feelings of guilt and marked stress after an episode. BED occurs without typical compensatory behaviors of bulimia nervosa that are targeted to prevent weight gain, such as self-induced vomiting, fasting, laxative misuse, or strenuous physical activity ( 1 ).

Data from the World Health Organization’s World Mental Surveys reveals BED as the most common eating disorder and a major public health problem, with a lifetime prevalence rate of 1.4%, ranging from 0.8% to 1.9% ( 2 ). BED is associated with psychiatric and physical comorbidities, including major depression and other psychiatric disorders ( 2 , 3 ), stress, impairment of social development ( 2 ), chronic pains ( 2 , 3 ), and diabetes mellitus ( 4 ).

BED is highly associated with obesity ( 2 , 3 , 5 ). In a group of adults with BED, 71% had a body mass index (BMI) ≥ 30 kg/m^2^ ( 6 ). More than 25% of children and adolescents with overweight and obesity have binge/loss of crontrol eating ( 7 ). BED is particularly common among those seeking treatment for weight reduction. Among candidates for bariatric surgery, it is the second most common psychiatric diagnosis, exceeded only by depressive disorder, but the prevalence is quite variable from 4% to 49% ( 8 ).

Individuals with obesity and BED have a higher frequency of irregular eating patterns, such as snacking, nibbling, and/or a tendency to double meals ( 9 ). Irregular eating behaviors in individuals with obesity and BED can interfere in the signaling of hormones that regulate the gut-brain axis appetite-signaling system (system related to the control of hunger and satiety) ( 10 ). Compared to people with obesity without BED, those with BED have higher levels of leptin ( 11 , 12 ). Individuals with obesity have, paradoxically, decreased serum ghrelin levels ( 13 ), and those with obesity and BED have even lower levels of ghrelin compared to individuals without the eating disorder ( 14 - 16 ). So far, no research has sufficiently clarified whether serum levels of GLP-1 are altered in obese and BED patients. In a study conducted by Geliebter and cols. ( 16 ), no differences were observed in fasting or post-prandial plasma levels of GLP-1 between obese groups with and without BED.

Studies performed in animal models have demonstrated that inflammation can also interfere in the control of hunger and satiety, changing afferent and efferent signaling related to the gut-brain axis by reducing sensitivity to ghrelin ( 13 ) and GLP-1 ( 17 ). Obesity is characterized by a low-grade chronic inflammatory state. Inflammatory cytokines can be produced within the central nervous system (induced by diet and stressors associated with food) and act directly on hypothalamic neurons involved in the regulation of food and eating behavior. Inflammatory cytokines are associated with obesity related to poorly adapted eating behavior ( 18 ). Inflammation may be a direct effect of binge eating, perhaps because of the large amount of food ingested in typical eating binges ( 19 ). Indeed, over nutrition causes enlarging adipocytes to change their intrinsic secretion profile towards a pro-inflammatory phenotype, characterized by a higher secretion of TNF-α ( 20 , 21 ). This pro-inflammatory cytokine can leak out of the adipose tissue and produce systemic effects ( 22 ). The low-grade chronic inflammation state associated with overweight and obesity may also interfere with food control by decreasing sensitivity to leptin ( 23 ).

We hypothesized that factors such as psychiatric comorbidity, dietary patterns, circulating levels of biological inflammatory markers, and hormones of the gut-brain axis could be influenced by the presence of BED in obese individuals. A comparison of such factors in BED and non-BED obese groups of patients represents a unique opportunity to clarify the relationship between inflammatory markers, hormones of the gut-brain axis, and BED. To the best of our knowledge, no studies have investigated all these factors jointly in the same individuals in a cohort. This study aimed to compare participants’ depressive and anxiety symptoms, circulating inflammatory markers, and hormonal peptides related to the gut-brain axis to identify differences between obese patients with and without BED.

## MATERIALS AND METHODS

### Patients and study design

We designed a cross-sectional study involving subjects with obesity who works or study in a university hospital. From December 2017 to December 2018, 284 adults of both sexes volunteered to participate in the study by responding to posters that had been distributed around the hospital. Inclusion criteria were: age > 20 years; BMI ≥ 30 kg/m² and ≤ 45 kg/m²; able to answer self-report questionnaires. Exclusion criteria were: diagnosis of diabetes mellitus; impaired thyroid function; known inflammatory disease; diagnosis of psychiatric disorder other than BED, anxiety or depression; history of malignant disease or pathologies; currently dieting or participating in a weight loss program; receiving hormonal therapy and/or pharmacological treatment with the potential to induce metabolic changes or cognitive impairment, such as antidepressants, anxiolytics and other psychotropic drugs; history of substance abuse or dependence. Smokers, post-menopausal women and people with a history of bulimia nervosa or compensatory behavior were also excluded. Participants provided their written informed consent, and the study was approved by the university hospital Research Ethics Committee (protocol CAAE 01526012.9.0000.527). All procedures were conducted in accordance with the Declaration of Helsinki principles.

### Psychiatric evaluation

A psychiatrist with expertise in the field of eating disorders interviewed each volunteer. The interviewer applied the Structured Clinical Interview for DSM-5 – Clinician Version (SCID-5-CV) ( 24 ), to assess/confirm the diagnosis of BED, and depression and anxiety disorders.

Patients completed a 16-item self-administered questionnaire, the Binge-Eating Scale, developed by Gormally and cols. ( 25 ) and translated and validated in its Brazilian version by Freitas and cols. ( 26 ). Each item consists of four options where the individual chooses from 0 to 3, expressing the severity of that item, reflecting their feelings about their eating behavior. A total BES index ≤ 17 indicates mild or no binge eating, an index of 18-26 indicates moderate binge eating, and an index ≥ 27 indicates severe binge eating. Patients were also required to fill out the self-report Hospital Anxiety and Depression Scale (HADS), which consists of 14 items scored on a 4-point Likert scale (range 0-3) ( 27 ) and has been translated and validated for use in Brazil ( 28 ). The total score is the sum of the 14 items; for each subscale, the score is the sum of the respective seven items (range 0-21).

### Eating pattern assessment

An experienced investigator in the field of eating disorders conducted an in-depth assessment of participants’ eating patterns, during the previous six months. The eating patterns included hyperphagia, bingeing, post-dinner eating, feeling “stuffed”, and emotional eating.

### Daily calories intake measurement

Food intake was estimated using the non-consecutive three-day food record method (two weekdays and one weekend day). The patients were taught by the investigators how to properly fill out the records. The average daily caloric intake was then calculated by entering the data from the patients´ food records in the software WebDiet Health Manager – version 3.0, Rio de Janeiro, Brazil.

### Anthropometric measures and body composition determination

BMI was calculated as weight (in kilograms) divided by height (in meters) squared. Waist circumference (WC) was measured in standing position at the level of the umbilicus. Bioelectrical impedance analysis (BIA) was undertaken with a tetrapolar bioanalyzer device (Model 310, Biodynamics Corp, Seattle, WA, USA) to estimate body composition, as previously described ( 29 ).

### Laboratory analysis for plasma cytokines and hormones determination

After 12 hours of fasting, blood samples were collected into prechilled 10 mL blood collection tubes that contained spray-dried K2EDTA anticoagulant. Immediately after collection, plasma was separated by centrifugation at 2,000 g for 15 min at 4 °C, then portioned in 2 mL aliquots into Eppendorf tubes and stored at -80 °C until assayed. Plasma concentrations of IL-6, ghrelin, TNF-α, leptin, and GLP-1 (Catalogue number HMHMAG-34K-05) were analyzed by multiple analysis profiling (MAP) through the multiplex TM xMAP technology (Merck Millipore, Brazil). Plasma CRP was analyzed by enzyme immunoassay (C-Reactive Protein Elisa Kit, Human, Merck, Millipore, Brazil). The laboratory assays were performed following the manufacturer’s instructions.

### Statistical analysis

Data are presented as means ± standard deviations (SD), numbers of participants, percentages, and odds ratios (OR) with 95% confidence intervals (95% CI). Categorical variables were compared by a *χ* ² test. The Kolmogorov-Smirnov test was used to evaluate the normal distribution of data sets. Student’s t-test or the Mann-Whitney U test was used to compare data among subjects. Binary logistic regressions were performed to assess independent factors as potential predictors for the presence of BED in the sample, after adjusting for gender and age. Input variables were depressive symptoms, leptin, GLP-1, CRP, and TNF-α. Statistical analyses were performed using the Statistical Package for the Social Sciences for Windows, version 16.0 (SPSS, Inc., Chicago, IL). Statistical significance was set at p < 0.05.

## RESULTS

### Sample characteristics and psychiatric evaluation

**Figure 1:**
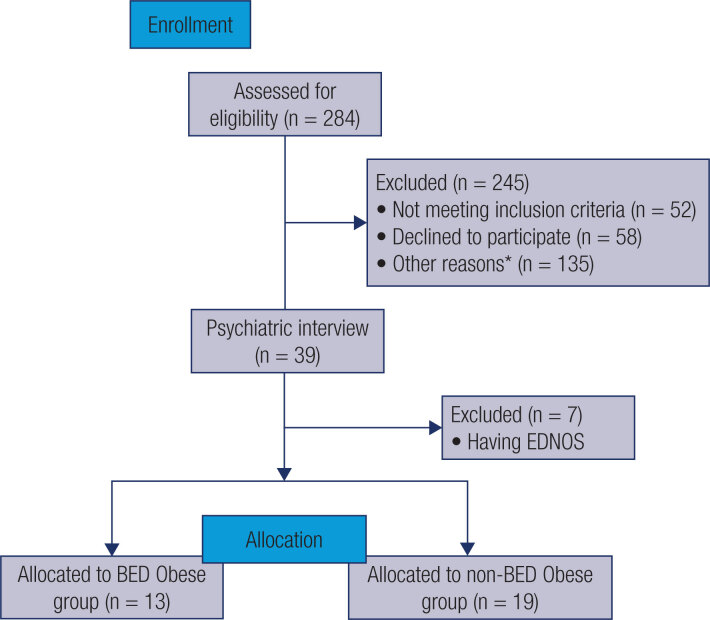
Study population flowchart diagram. EDNOS: eating disorder not otherwise specified. *Women in postmenopause (n = 57); diabetes (n = 22); hypo- or hyperthyroidism (n = 21); inflammatory diseases (cardiomyopathies, systemic lupus erythematosus, endometriosis, cancer, multiple sclerosis, venous thrombosis, systemic arterial hypertension, n = 20); use of psychotropics (neuroleptics, antidepressants and mood stabilizers, n = 12); smoking (n = 2); use of corticosteroids (n = 1).

Of the 284 adults that volunteered to participate in the study, 245 were excluded because of the following reasons: not meeting inclusion criteria for IMC (n = 46), age (n = 3) or ability to answer self-report questionnaires (n = 3); women in postmenopause (n = 57); diabetes (n = 22); hypo- or hyperthyroidism (n = 21); inflammatory diseases (cardiomyopathies, systemic lupus erythematosus, endometriosis, cancer, multiple sclerosis, venous thrombosis, systemic arterial hypertension, n = 20); use of psychotropics (neuroleptics, antidepressants and mood stabilizers, n = 12); smoking (n = 2); use of corticosteroids (n = 1); and declined participation (n = 58). After these exclusions, 39 volunteers continued the screening process and were submitted to a psychiatric evaluation ( Figure 1 ). At this point, seven volunteers (18%) were excluded because they were identified as having EDNOS (Eating Disorder Not Otherwise Specified). Moreover, the psychiatric interview (SCID-5-CV) identified 13 individuals (33%) as having BED, and 19 non-BED individuals (49%).

Thereby, 32 subjects fulfilled eligibility criteria ( Figure 1 ). They were mainly hospital employees or their close acquaintances. According to the psychiatric interview (SCID-5-CV), they were divided into two study groups: the BED group (n = 13) and the non-BED group (n = 19) ( Figure 1 ). Table 1 shows the main socio-demographic, anthropometric, and clinical characteristics of study participants from these two groups. No intergroup differences were found regarding socio-demographic and anthropometric variables such as age, schooling, BMI, abdominal circumference, fat mass, and lean mass ( Table 1 ).

**Table 1:** Socio-demographic, anthropometric, and clinical characteristics of non-BED and BED groups

	Non-BED group						BED group						p
	Mean	±	SD	Median	Min.	Max.	Mean	±	SD	Median	Min.	Max.	
n (man/woman)	19		(2/17)	-	-	-	13		(1/12)	-	-	-	-
Age (years)	38.1	±	8.0	37.0	27	56	33.0	±	6.7	34.0	23	46	0.69
Schooling (years) †	8.5	±	2.8	9.0	4.0	12	8.1	±	2.9	9.0	4.0	12	0.70
Body mass index (kg/m^2^)	36.3	±	4.1	35.7	30.5	43.6	36.2	±	2.1	37.3	33.1	38.5	0.90
Abdominal circunference (cm)	109.0	±	14.2	107.5	80.5	137.5	105.4	±	9.3	104	90	127	0.41
Fat mass (%)	37.5	±	2.8	37.7	33.4	41.9	39.4	±	8.4	37.6	32.7	66.2	0.37
Lean mass (kg)	59.2	±	13.0	58.7	27.7	89.5	60.9	±	6.4	61.7	48.6	73.7	0.65
Anxiety symptoms (scores)	6.2	±	3.6	6.0	0	13	8.5	±	4.4	8.0	3	18	0.05
Depressive symptoms (scores)	12.2	±	6.7	12.0	2	23	17.4	±	7.4	16.0	9	34	**0.04**

† Variables converted into logarithms for statistical analysis. BED: binge eating disorder. Schooling was tested with the Mann-Whitney Test, all other variables were tested with Student’s *t* -test.

*p* < 0.05: statistically significant.

When compared to the non-BED participants, the BED group showed significantly higher mean scores for depressive symptoms (17.4 ± 7.4 *vs.* 12.2 ± 6.7 points, p = 0.04), and higher scores of anxiety symptoms with a marginal significance level (8.5 ± 4.4 *vs.* 6.2 ± 3.6 points, p = 0.05), according to HADS ( Table 1 ). The BED group had a higher frequency of depressive disorder than the non-BED group, with a marginal significance level (30.8% *vs.* 5.3%, *χ* ^2^ = 3.76, p = 0.05). There were no statistically significant differences on frequency of anxiety, even though there is a 3x higher frequency of anxiety in the BED group compared to non-BED group (46.2% *vs.* 15.8, *χ* ^2^ = 3.521, p = 0.06), according to SCID-5-CV.

### Eating patterns of BED and non-BED obese participants

**Figure 2:**
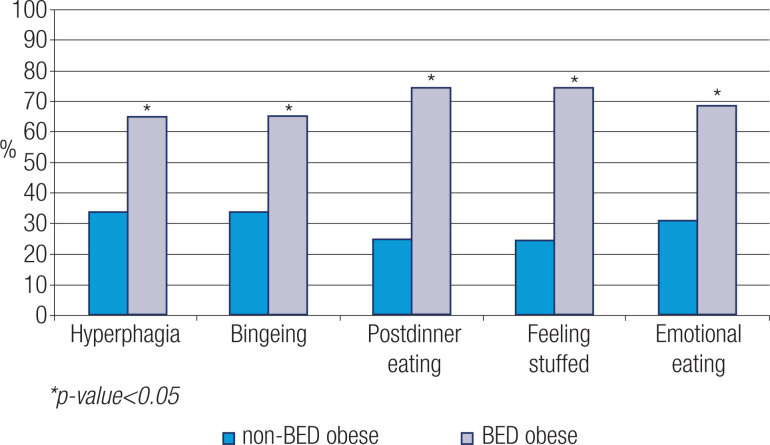
Eating patterns in non-BED and BED obese patients. The asterisks indicate values of *p* obtained by Pearson´s chi-squared test.

Regarding eating patterns, the BED obese group revealed significantly higher percentages of hyperphagia, bingeing, post-dinner eating, feeling “stuffed”, and emotional eating than the non-BED obese participants ( Figure 2 ). No significant differences were observed in daily caloric intake between groups with or without BED (1850 ± 619.36 cal/day *vs.* 2253.12 ± 827.72 cal/day; p = 0.146).

### Hormonal and inflammatory profiles and multivariate analysis of factors possibly associated with BED

Evaluation of hormonal and inflammatory profiles in plasma samples revealed that leptin levels were 33% higher in the BED group than in the non-BED group (p = 0.01). GLP-1 levels were 89% higher in the BED group (p = 0.02). Serum CRP levels were 67% higher (p = 0.01), and TNF-α levels were 53% higher (p = 0.01) in the BED group compared to the non-BED group. There was no difference in ghrelin (p = 0.97) and IL-6 (p = 0.50) levels between the groups ( Table 2 ).

**Table 2:** Comparison of hormonal and inflammatory profile between non-BED and BED groups

	Non-BED group						BED group						p
	Mean	±	SD	Median	Min.	Max.	Mean	±	SD	Median	Min.	Max.	
Leptin (pg/mL)	25.8	±	7.7	25.7	11.5	38.6	34.3	±	11.3	31.2	12.7	50.1	**0.01**
Glucagon-like peptide 1 (pg/mL) †	27.4	±	14.7	25.9	11.0	55.9	51.9	±	38.5	32.4	14.5	134	**0.02**
Ghrelin (pg/mL) †	6.8	±	2.9	5.4	4.0	13.6	6.8	±	6.9	4.1	4.0	28.9	0.97
C reactive protein (µg/mL)	16.5	±	10.1	16.3	2.9	40.0	27.5	±	14.6	23.5	9.9	62.2	**0.01**
Tumor necrosis fator α (pg/mL)	3.6	±	2.5	4.0	0.6	9.0	5.5	±	1.5	6.0	1.9	7.0	**0.01**
Interleukin 6 (pg/mL) †	5.0	±	6.2	3.1	0.9	21.9	5.0	±	8.3	2.4	0.9	31.4	0.50

† Variables converted to logarithms for statistical analysis. BED: binge eating disorder. Ghrelin was tested with the Mann-Whitney test, all other variables were tested with the Student’s t-test.

*p* < 0.05: statistically significant.

Possible predictors of the presence of BED were evaluated through logistic regression models performed with stepwise selection. Only the statistically significant variables presented in Table 3 entered in the model (depressive symptoms, leptin, GLP-1, CRP, TNF-α). Increased depressive symptoms (p = 0.031) and elevated CRP (p = 0.024) were independently associated with BED ( Table 3 ).

**Table 3:** Binary logistic regression to assess factors associated with binge eating disorder

Outcome	Significant variable	Beta	S.E.	Odds ratio	95% CI	*p* value
Presence of BED	Depressive symptoms	0.160	0.074	1.173	1.015-1.356	**0.031**
	C reactive protein	0.115	0.051	1.122	1.015-1.240	**0.024**

S.E: standard error; CI: confidence interval; BED: binge eating disorder. n = 32. *p* < 0.05: statistically significant.

## DISCUSSION

In this study, we compared a range of inflammatory markers, appetite-related hormones levels, and symptoms of depression and anxiety in individuals with obesity with and without BED. The presence of depressive symptoms and plasma levels of CRP, TNF-α, leptin, and GLP-1 were altered in individuals with BED compared to those without BED. Moreover, depressive symptoms and CRP plasma levels were independently associated with the presence of BED.

In the present study, which included volunteers with obesity who were not seeking psychiatric, nor obesity treatment, the frequency of BED was 33%. The frequency of BED varies according to the type of sample and the diagnostic criteria used for its assessment ( 30 ). The reported lifetime prevalence of BED in community samples is 1,4% ( 2 ). While around 4.5% of individuals with BMI ≥ 35 meet criteria for BED ( 31 ), BED prevalence among candidates for bariatric surgery rates between 4% to 49% ( 8 ). Although the frequency of BED reported in the present study within individuals with BED and obesity not seeking treatment seems to be high when compared to the above-cited studies, others reported samples with approximately 50% of BED frequency among individuals with overweight and obesity ( 32 , 33 ). We hypothesize that discrepancies may be attributable to different methods and criteria used to assess the diagnosis of BED in addition to differences in sample size.

All participants in our study who had BED also had depressive symptoms. Depressive scores were 50% higher in the BED group than in the non-BED group. Increased rates of depressive symptomatology are consistent with previous studies that have shown a greater frequency of psychiatric disorders in individuals with obesity and BED than in those without the disorder ( 32 , 34 ). Our results corroborate the findings reported in the literature that the degree of depressive symptomatology evidenced in individuals with obesity and BED is related to the severity of binge eating, not to the severity of obesity ( 34 ). Moreover, individuals with obesity and depression associated with BED tend to eat more in response to negative emotions and anxiety, which correlates with binge eating and most eating psychopathology measures, putting them at greater risk of developing and maintaining obesity ( 35 , 36 |).

Similar to findings observed in other studies, the BED group in the present research showed a higher frequency of altered eating patterns, such as emotional eating, bingeing, hyperphagia and post-dinner eating ( 37 - 39 ).

In this study, low-grade systemic inflammation was indicated by higher circulating levels of CRP and TNF-α found in the BED group in comparison with the non-BED group. Our results are in line with those of Succurro and cols. ( 39 ), who reported that individuals with obesity and BED have higher inflammatory markers compared to obese individuals without BED. Our results revealed that depressive symptoms and CRP levels are independent factors related with BED. Depression confers a higher risk for developing BED ( 40 ). In the presence of both BED and depression there is a greater tendency to eat in response to emotions ( 35 , 36 ). As all the BED patients showed depressive symptoms, it could be hypothesized that the higher inflammatory status among BED patients is associated to depressive disorders spectrum. Extensive studies have discussed evidence on the associations between immune-related processes and depressive symptoms, suggesting a role of inflammation in the etiology of depression ( 41 - 44 ). Pro-inflammatory markers, such as CRP, TNF-α, IL-1 and IL-6, have been found at increased levels among depressed subjects ( 45 - 48 ). In addition, elevated levels of CRP are associated with increased risk for development of depression in the general population ( 49 ). Therefore, we tested whether a causal relationship exists between depression and inflammation in subjects with BED through regression analysis; however, we did not find a significant association between depressive symptoms and inflammatory markers either in the BED group or in the total sample. Future studies linking obesity, depressive symptoms and inflammatory profile with large sample size must still be undertaken to better elucidate whether inflammation contributes to the occurrence of depression in individuals with BED and obesity and if the immune system can be targeted to effectively prevent and treat depression and BED in individuals with obesity.

In the present study, leptin levels were 33% higher among patients with BED. These results are similar to those found by Adami and cols. ( 12 ), who reported increases of 28% in leptin levels among obese individuals with BED, when compared to those without the disorder, in a sample comprised of morbidly obese individuals. It has been suggested that, for women with BED who exhibit increased levels of leptin, impaired sensitivity to the enhanced circulating leptin could be involved in the pathogenesis and/or maintenance of binge eating ( 11 ). In individuals with leptin resistance, the brain’s response becomes blunted, and leptin no longer produces the same degree of satiety after a meal ( 10 ). Furthermore, leptin resistance may represent an underlying mechanism linking obesity with depression with atypical features, like hyperphagia ( 50 ). The negative results found by others ( 14 ) can be explained by the lower degree of obesity in the participant samples since leptin levels vary according to fat percentage and BED ( 12 ).

In the present study, no significant differences were observed in ghrelin levels between groups with or without BED. Similar results were found by other authors ( 15 , 51 ). Differences in ghrelin plasma levels reported by different researchers may be due to participants’ sex, grade of obesity, and the time of day and fasting periods for blood collection ( 52 ).

We found higher levels of the satiety-promoting hormone GLP-1 in the BED group than the non-BED group. To this date, only one study examined the levels of GLP-1 in obese BED and non-BED women and did not find any differences between the studied groups ( 16 ). It has been reported that GLP-1 secretion can be increased by inflammatory stimuli ( 53 ). In addition, Anini and cols. ( 54 ) reported that leptin stimulates GLP-1 secretion both in human intestinal endocrine L-cells *in vitro* in a dose-dependent manner, and in an obese mouse model. Therefore, both pro-inflammatory cytokines and leptin could play a role in stimulating GLP-1 production. In the present study, both leptin and CRP levels were higher among patients with BED. From our point of view, increased levels of GLP-1 in individuals with BED could be influenced by the greater inflammation and higher levels of leptin found in this group.

Moreover, it has been recently discussed the promising beneficial roles of GLP-1 in alleviating depression through the regulation of neuroinflammation, synaptic dysfunction and impaired neurogenesis and neurotransmitter secretion, which are key processes known to be involved in the pathogenesis of depression ( 55 , 56 ). Therefore, we hypothesize that increased levels of GLP-1 in individuals with BED – which were all found with depressive symptoms – could be a response to neuronal dysfunctions in the depressive brain. This emphasize the need of further studies concerning the mechanisms and function of GLP-1 in the context of BED, depression and obesity, taking into account the neuro-immune-endocrine complex aspects of these diseases.

Limitations of this study include the nature and size of the sample, which may limit generalizability. The exclusion of patients using psychotropics could be considered as a limitation of the study, because severe psychiatry patients could have been excluded from the analysis. However, some of the psychotropics are known to be responsible for metabolic changes, such as hyperglycemia and weight gain. Therefore, taking psychotropics could be an external metabolic disruptor, acting as a confounding variable to evaluate if the presence of BED is associated to the level of metabolic hormones. Owing to its cross-sectional design of the study, a causal relationship cannot be definitively established. Another limitation of our study is that our findings refer to a single morning measurement of blood hormones despite the well-recognized circadian rhythmicity in the secretion of these substances.

This study’s strengths include the exclusion of patients with confounding disorders characterized by elevation in inflammatory markers. The study also benefits from the accurate anthropometric and psychiatric characterization.

Results of the present study may have important clinical implications. Our data reinforce the idea that BED should be assessed as an indicator of clinical severity in all patients with obesity. Although BED is a relatively common eating disorder ( 57 ), only a small proportion of patients with BED are treated ( 58 ), and so, BED sufferers are underrecognized by the health care system ( 59 ). Binge eating in individuals with obesity is generally neglected and treatment focuses on obesity instead of addressing eating psychopathology ( 60 ).

In conclusion, in individuals with obesity, those with BED showed greater psychiatric comorbidity, worse eating patterns, worse inflammatory profile, and higher circulating leptin and GLP-1 levels than those without BED. Depressive symptoms and inflammation were independently associated with BED. These findings points to the relevance of the clinical assessment of eating behaviors and BED in individuals with obesity by a multidisciplinary team in order to help guide treatment.
